# Astroglia’s role in synchronized spontaneous neuronal activity: from physiology to pathology

**DOI:** 10.3389/fncel.2025.1544460

**Published:** 2025-03-19

**Authors:** Aswathy Ammothumkandy, Alisha Cayce, Mohammad Shariq, Michael A. Bonaguidi

**Affiliations:** ^1^Department of Stem Cell Biology and Regenerative Medicine, Keck School of Medicine, Eli and Edythe Broad Center for Regenerative Medicine and Stem Cell Research, University of Southern California, Los Angeles, CA, United States; ^2^Keck School of Medicine, Neurorestoration Center, University of Southern California, Los Angeles, CA, United States; ^3^Department of Neurological Surgery, Keck School of Medicine, University of Southern California, Los Angeles, CA, United States; ^4^Department of Biomedical Engineering, Viterbi School of Engineering, University of Southern California, Los Angeles, CA, United States; ^5^Department of Gerontology, University of Southern California, Los Angeles, CA, United States; ^6^Keck School of Medicine, Zilkha Neurogenetic Institute, University of Southern California, Los Angeles, CA, United States

**Keywords:** epilepsy, astrocyte, glia, neuronal activation, cognition, mental health, sleep, epileptogenesis

## Abstract

The nervous system relies on a balance of excitatory and inhibitory signals. Aberrant neuronal hyperactivity is a pathological phenotype associated with several neurological disorders, with its most severe effects observed in epilepsy patients. This review explores the literature on spontaneous synchronized neuronal activity, its physiological role, and its aberrant forms in disease. Emphasizing the importance of targeting underlying disease mechanisms beyond traditional neuron-focused therapies, the review delves into the role of astroglia in epilepsy progression. We detail how astroglia transitions from a normal to a pathological state, leading to epileptogenic seizures and cognitive decline. Astroglia activity is correlated with epileptiform activity in both animal models and human tissue, indicating their potential role in seizure induction and modulation. Understanding astroglia’s dual beneficial and detrimental roles could lead to novel treatments for epilepsy and other neurological disorders with aberrant neuronal activity as the underlying disease substrate.

## 1 Introduction

**FIGURE 1:**
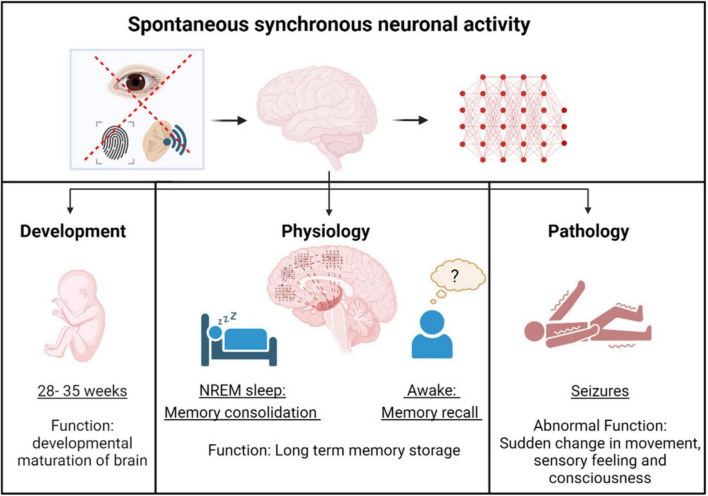
Spontaneous synchronous neuronal activity in development, physiology and pathology. Spontaneous synchronous neuronal activity occurs in the brain during the pre-birth stage, facilitating the developmental maturation of neural circuits. After birth, neuronal activity becomes more non-spontaneous, stimulus-evoked, and spatially restricted, except during free memory recall and memory consolidation in non - rapid eye movement (NREM) sleep. Under pathological conditions, aberrant spontaneous synchronous neuronal activity, known as epileptiform activity, leads to seizures.

Nervous system function is mediated by a proper balance of excitatory and inhibitory signals. However, abnormal neuronal hyperactivity is a key feature in normal aging (Zullo et al., 2019) as well as several pathological conditions including epilepsy (Engel, 2001), Alzheimer’s Disease (Lam et al., 2017), Glioma (Snijders et al., 2017) and even severe SARS-CoV2 infection (Lin et al., 2021). In addition to causing debilitating seizures, neuronal hyperactivity is associated with progressive cognitive decline in patients with epilepsy (Coan and Cendes, 2013), as well as subclinical epileptiform activity (Vossel et al., 2016). In the first part of this review (see section “2.1 Synchronized spontaneous neuronal activity in the developing brain”–“2.3 Synchronized spontaneous neuronal activity in pathology”), we summarize the current literature for synchronized spontaneous neuronal activity in healthy physiological state and further discuss its aberrant forms in pathology (Figure 1). In the second part (see section “3.1 Epileptogenesis and key cellular events,” “3.2 Expanding beyond neurons: a neuronal centric view on human epilepsy drug development and the need for broader perspectives”) we discuss the underlying pathological changes and key cellular players that trigger sudden aberrant neuronal hyperactivity in the brain. In the third part of this review (see section “4.1 Astrocytes function in homeostatic neuronal activity”–“4.7 Astroglia in epilepsy associated mental health comorbidities”), we address the potential mechanisms by which astroglia transitions from a physiological to pathological state and how it could lead to triggering seizures through integration of spatiotemporal signals and later aggravating progressive cognitive impairment via chronic inflammation. Studies using mouse and zebrafish models have identified surges in astroglial activity prior to seizure activity (Diaz Verdugo et al., 2019; Tian et al., 2005). Our recent research using hippocampal tissue from human epilepsy surgical resections, shows that human immature astroglia activity anti-correlates with neuronal hyperactivity (Ammothumkandy et al., 2022). There is growing evidence that suggests astroglia’s role in neuronal network rhythmic activity and synchronization, mediating sudden behavioral state switches in physiology (Oliveira and Araque, 2022). Understanding how astroglia spatiotemporally integrate neuronal activity for influencing physiological functions and its evolution in disease progression could provide a novel therapeutic target for epilepsy as well as other pathological conditions with epileptiform activity. Most of the current anti-epileptic drugs are targeted at neuronal cells and therefore identifying novel cellular targets to prevent seizures as well as cognitive co-morbidities can have huge clinical impact.

## 2 Synchronized spontaneous neuronal activity in physiological and pathological brain

### 2.1 Synchronized spontaneous neuronal activity in the developing brain

Spatiotemporally fine-tuned sparse electrical activity between neurons is important for the normal function of the adult nervous system. However, in the prenatal stages the neuronal cells show highly synchronized spontaneous activity which plays a key role in early developmental processes like neurogenesis, migration, and formation of synaptic connections (Luhmann et al., 2016; Molnár et al., 2020). Studies in rodent models have shown that during early prenatal days, sensory-evoked excitatory and inhibitory signals are less correlated, but as experience-dependent refinement of intracortical inhibition occurs, excitation and inhibition becomes highly correlated (Dorrn et al., 2010). In the first couple of prenatal weeks neural activity transforms from a synchronized, dense oscillatory signal to sparse signals which is either dependent or independent of sensory signals (Golshani et al., 2009; Rochefort et al., 2009). This attainment of sparse neural signals is considered to be important in efficient encoding of sensory inputs in the mature brain (Olshausen and Field, 2004). Similarly in humans, electroencephalography (EEG) recordings in preterm babies have identified spontaneous and transient neuronal activities in the prenatal stage which declined with the time of normal birth (Vanhatalo et al., 2005). Further studies have combined data from EEG and Magnetic resonance imaging (MRI) to show that this increased early spontaneous activity transients in the preterm babies correlated with a faster growth of brain structures until the time of normal birth (Benders et al., 2015). The decline of spontaneous activity transients occurred in parallel with the developmental maturation of functional Gamma-aminobutyric acid (GABA) mediated inhibition (Vanhatalo et al., 2005). Therefore, both studies in rodents and humans show that there is a fine tuning of electrical activity that occurs during early prenatal and postnatal developmental stages.

### 2.2 Synchronized spontaneous neuronal activity in the adult brain

During early postnatal stages, neuronal activity becomes more spatially restricted during wake states, while spontaneous large-scale waves propagate across the cortex during sleep cycles (Tabuena et al., 2022), a physiological process which continues into adulthood. In adults, the synchronized spontaneous neuronal oscillations are specifically observed during the non - rapid eye movement (NREM) stage of sleep and are characterized by high amplitude slow waves (< 1 Hz), delta waves (1–4 Hz) and sleep spindles (12–16 Hz) (Brown et al., 2012; Dang-Vu et al., 2008; Steriade et al., 1994). These high levels of synchronized activity (denoted by high amplitude) during NREM sleep are critical in consolidation of memory from hippocampus into the neocortex (Geva-Sagiv et al., 2023; Helfrich et al., 2019; Rasch and Born, 2013). During NREM sleep cycle, high frequency (200 Hz) spontaneous network oscillations called sharp wave ripples are observed in the hippocampus (Buzsáki et al., 1992). Recently, spontaneous synchronized sharp wave ripples in the hippocampus have also been observed in awake states in the adult human brain in association with spontaneous free-memory recall (Norman et al., 2019). There is also a synchrony of spontaneous high frequency oscillations in the hippocampus and cortex, which is considered critical for binding memory about different aspects of an event across different cortical regions (Dickey et al., 2022). Overall, spontaneous synchronized neuronal network activity is a physiological process necessary for memory consolidation. It is extensively studied in relation to normal sleep cycles and is emerging as important for cognitive functioning during wakefulness. Further research is needed to fully understand its functional significance and the underlying mechanisms that distinguish it from non-spontaneous, stimulus-evoked neuronal activity.

### 2.3 Synchronized spontaneous neuronal activity in pathology

Abnormal synchronized spontaneous neuronal hyperactivity is a pathological hallmark of several neurological disorders such as epilepsy (Engel, 2001), Alzheimer’s Disease (Lam et al., 2017; Vossel et al., 2016), Glioma (Snijders et al., 2017), and Huntington’s disease (Landau and Cannard, 2003). The aberrant electrical activity creates an imbalance in the nervous system’s homeostatic ability to fine tune human behavior. This leads to sudden changes in movement, consciousness, and sensory feelings which are called seizures (Devinsky et al., 2018). This aberrant electrical activity in the brain during a seizure can be detected by EEG and is called epileptiform activity, characterized by brain waves with altered amplitude and frequency patterns (Fisher et al., 2014; Wang and Mengoni, 2022). In certain neurological conditions the patient does not have a seizure, but EEG recordings of the brain detect aberrant electrical activity which is classified as sub clinical epileptiform activity. Both epileptiform activity (Coan and Cendes, 2013) as well as subclinical epileptiform activity (Horváth et al., 2018; Vossel et al., 2016) progressively leads to cognitive decline. In addition to neurological disorders, neuronal hyperactivity is also associated with aging (Zullo et al., 2019), and might be a potential substate for age associated cognitive decline.

## 3 Underlying pathological changes and key cellular players in epilepsy

### 3.1 Epileptogenesis and key cellular events

**FIGURE 2:**
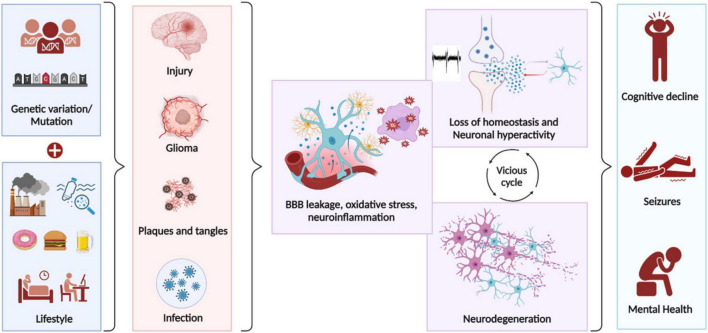
Key cellular events that lead to epileptogenesis. Genetic mutations and lifestyle factors influence the brain’s vulnerability to undergo pathological changes following brain insults, potentially leading to epilepsy. The progression to epilepsy occurs over a latency period during which numerous cellular and molecular changes accumulate. A common cascade of aberrant cellular events that occur during epileptogenesis, regardless of etiology includes Blood brain barrier (BBB) leakage, oxidative stress and neuroinflammation. These events disrupt homeostasis, cause abnormal circuit development, and lead to seizures. This non-homeostatic state perpetuates a vicious cycle that can chronically cause neurodegeneration, resulting in cognitive decline and mental health issues.

The progression of a balanced homeostatic brain to an epileptic brain is called epileptogenesis (Pitkänen and Lukasiuk, 2011). Epilepsy is either caused by genetic mutations (Noebels, 2015) leading to abnormal circuit development or is acquired by a brain insult (Devinsky et al., 2018). Stroke, traumatic brain injury, infection, tumor, neurodegenerative condition, and other unknown factors can act as brain insults that lead to acquired forms of epilepsy. Currently lifestyle changes have not shown to be a direct cause for epilepsy; however, factors like alcohol, unhealthy diet, pollutants, poor sleep, lack of exercise are all factors that increase the vulnerability of other brain disorders (Kip and Parr-Brownlie, 2023; Popa-Wagner et al., 2020) which can then potentially lead to epilepsy as a secondary event. Brain insult leading to epilepsy occurs over a latency period during which several cellular and molecular changes accumulate within the brain leading to imbalance in excitatory and inhibitory signals (Herman, 2002). A cascade of aberrant cellular events that are common to all these brain disorders and the subsequent acquired epilepsy (Klein et al., 2018) are Blood brain barrier (BBB) leakage (Gorter et al., 2019; Knox et al., 2022), oxidative stress (Fesharaki-Zadeh, 2022; Patel, 2002; Zilberter et al., 2022) and neuroinflammation (Chen Y. et al., 2023; Mishra et al., 2021; Simon et al., 2017). Further it leads to loss of homeostasis in the fine tuning of neuronal activity leading to seizures (Casillas-Espinosa et al., 2012). This non-homeostatic state is maintained in a vicious cycle that can chronically cause neurodegeneration (Casillas-Espinosa et al., 2020), which then progressively leads to cognitive decline and mental health issues (Holmes, 2015; Figure 2).

In response to brain insult, the neuroinflammatory pathways can be triggered immediately by extracellular inflammation mediators released by necrotic cells (Scaffidi et al., 2002). Recent study has shown that even acute neuroinflammation disrupts neuronal chloride regulation, which leads to loss of GABAergic inhibition and neuronal hyperexcitability (Kurki et al., 2023). Neuroinflammation over time activates microglia and astrocytes to pro-inflammatory state leading to a positive feedback loop of excess pro-inflammatory cytokine production which further leads to epileptogenesis (Chen Y. et al., 2023; Vezzani et al., 2011). The neuroinflammatory pathways can also result in BBB disruption, oxidative stress, and pathogenesis (Evran et al., 2020). BBB disruption leads to serum protein exposure in the brain contributing to epileptogenesis (Seiffert et al., 2004). The exposure to albumin results in astrocyte dysfunction, impairing its ability to clear excess potassium and glutamate causing neuronal hyperexcitability (David et al., 2009). In human Temporal lobe Epilepsy (TLE), albumin extravasation was observed in the astrocytes and neurons of epileptic hippocampus compared to control autopsies and the levels were highest in association with status epilepticus (van Vliet et al., 2007). Brain insult, neuroinflammation and BBB disruption can all lead to oxidative stress in neurons and glia which also augments the pathophysiological process involved in epileptogenesis (Fesharaki-Zadeh, 2022; Patel, 2002). Oxidative stress is observed both in rodent epilepsy models as well as human hippocampi post-status epilepticus. Transient treatment with antioxidant drugs during epileptogenesis in rats significantly reduced oxidative stress, delayed epilepsy onset, blocked disease progression, reduced seizure frequency, decreased hippocampal neuron loss, and rescued cognitive deficits (Pauletti et al., 2019). The collective effects of neuroinflammation, BBB break down, oxidative stress and loss of homeostatic neuronal activity leads to neuronal cell death and neurodegeneration (Farrell et al., 2017). Neuronal cell death can then feed to the vicious cycle of neuroinflammation and thereby epilepsy progression (Scaffidi et al., 2002). This might explain the progressive decline of cognition that is observed in epilepsy patients (Hermann et al., 2007) as well as the shared pathology between epilepsy and neurogenerative disorders (Negi et al., 2023; Targa Dias Anastacio et al., 2022). Inflammation is also known to detrimentally effect learning and memory by negative regulation of adult neurogenesis (Ekdahl et al., 2003; Jessberger et al., 2009; Monje et al., 2003; Shariq et al., 2021; Villeda et al., 2011). Similarly, in addition to loss of neurons, seizures can also result in cognitive decline by depletion of neuro stem cells and altered cell fate from neurogenesis to astrogenesis (Aimone et al., 2010; Fu et al., 2019; Sierra et al., 2015). Our recent studies in human MTLE showed that with increase in epilepsy disease duration there is a decline in neurogenesis (Ammothumkandy et al., 2022), which also linked with cognitive decline (Ammothumkandy et al., 2025). Therefore, the progressive neurodegeneration that occurs in epilepsy is attributed to both increase in neuronal cell death and a reduction in neurogenesis. Collectively epileptogenesis involves a complex interplay of non-homeostatic cellular and molecular changes leading to seizures and cognitive decline. While neurons have been the primary focus in epilepsy treatments, emerging evidence establishes the pivotal role of underlying disease mechanisms, which opens new avenues for therapeutic interventions.

### 3.2 Expanding beyond neurons: a neuronal centric view on human epilepsy drug development and the need for broader perspectives

Within the past 150 years there have been three generation of anti-epileptic drugs (AED) that have reached epileptic patients and/or have passed the FDAs drug safety and efficacy requirements. All three generations of AEDs target either inhibitory or excitatory neurons to mitigate onset and severity of seizures (Löscher and Schmidt, 2011; Sánchez et al., 2024). This is because Glutamatergic neurons have been known to play the central role in initiating seizure onset, which is caused by disharmony between excitatory and inhibitory cellular mechanisms (Barker-Haliski and Steve White, 2015). Glutamate is the most common form of excitatory neurotransmitter, and its intracellular build up plays an important role in epileptogenesis and cumulative excitotoxicity. A nefarious cycle of deviant neuronal connectivity and sporadic depolarization leads to higher glutamate in the post synaptic cleft leading to seizures. Therefore, the focus of AED drug development has been on reducing the excitatory function of glutamatergic neurons directly, or indirectly by increasing the function of inhibitory neurons (Corrales-Hernández et al., 2023; Löscher and Schmidt, 2011). There are approximately 30 approved AEDs currently, yet roughly thirty percent of epileptic patients are still refractory to these treatments and necessitate surgical resectioning of their focal epileptic tissue in order to obtain seizure freedom (Löscher and Schmidt, 2011). Though the AEDS provide moderate seizure control in about 70% of patients, despondently 60% of patients experience adverse side effects (Hanaya and Arita, 2016). Identifying new AEDs that target the underlying disease mechanisms and epileptogenesis is critical for (1) treating pharmacoresistant seizures, (2) avoiding adverse AED side effects, (3) curing epilepsy seizures, cognitive and mental health co-morbidities. There is a significant gap in AED targeting of non-neuronal cell types, with virtually no AED specifically targeting astrocytes. This is despite extensive research demonstrating the role of astrocytes in modulating physiological neuronal activity as well as pathological neuronal hyperexcitability, contributing to seizure onset and disease progression (Vezzani et al., 2022).

## 4 Astroglia transitions from a physiological to pathological state leading to epilepsy progression

### 4.1 Astrocytes function in homeostatic neuronal activity

Normal neuronal excitability relies on a fine balance between excitation and inhibition (E/I ratio), which is classically believed to be regulated by neuronal synaptic plasticity (Bhatia et al., 2019). Historically, astrocytes were thought to play a supportive role, but recent research has revealed their crucial functional role in the regulation of neuronal activity, synaptic plasticity, learning and memory. N-methyl-D-aspartate receptors (NMDARs) are essential ionotropic receptors that play significant roles in both developing and adult brain functions (Adesnik et al., 2008; Åmellem et al., 2021; Mu et al., 2015). NMDARs are known to influence two key postsynaptic receptors, alpha-amino-3-hydroxy-5-methyl-4-isoxazolepropionic acid receptor (AMPAR) and GABA type A receptor (GABAAR), leading to the regulation of the excitation-to-inhibition balance in neurons (Adesnik et al., 2008; Lu et al., 2011; Marsden et al., 2007; Muir et al., 2010). This E/I balance is vital for proper brain development, as well as for maintaining homeostatic neuronal activity in the adult brain. NMDARs control neuronal excitability by modulating the intracellular calcium influx upon activation, and their dysregulation is linked to a spectrum of neuropsychiatric and neurodegenerative disorders such as autism (Won et al., 2012), epilepsy (Sadeghi et al., 2021), Alzheimer’s (Olajide et al., 2021) and schizophrenia (Gilmour et al., 2009; Krystal et al., 2002). Astrocytes regulate extrasynaptic glutamate to modulate the activation of extrasynaptic NMDARs, which eventually leads to the regulation of synaptic transmission. Astrocytes can enhance synaptic efficacy through NMDAR activation via the secretion of ligands like glutamate and D-serine (Oliveira and Araque, 2022). A wide variety of neurotransmitters influence internal astrocytic calcium levels, which in turn results in the release of neuroactive molecules termed gliotransmitters, which modulate synaptic transmission and neuronal activity (Lines et al., 2020). Astrocyte-interneuron interactions are also known to regulate neural network activity within the hippocampus (Perea et al., 2016). Astrocytes exhibit both GABAergic and GABAceptive properties, playing a vital role in maintaining GABA homeostasis at intra and extracellular levels (Le Meur et al., 2012; Liu S. H. et al., 2022; Yoon et al., 2012). They primarily express the GABA transporters GAT1 and GAT3, which are crucial for modulating tonic inhibitory currents at the post synapse (Kersanté et al., 2013; Melone et al., 2015; Schousboe et al., 2017). Through GAT1 and GAT3 mediated GABA regulation, astrocytes influence synaptic efficacy and intracellular Ca^2+^ dynamics (Doengi et al., 2009; Matos et al., 2018; Parpura and Verkhratsky, 2012). Additionally, they contribute to BBB regulation by modulating vasodilation and vasoconstriction (Liu S. H. et al., 2022). Dysregulation of astrocytic GABA has been implicated in several neurological disorders, including Alzheimer’s disease (Brawek et al., 2018), epilepsy (Eid et al., 2013; Müller et al., 2020), and Parkinson’s disease (Roberts et al., 2020). The complex regulation of neuronal activity by astrocyte mediated regulation of ions and neurotransmitters is further reinforced by astrocytic morphological dynamics. Hormones like oxytocin (Theodosis and Poulain, 2001), norepinephrine (NE) (Sherpa et al., 2016; Vardjan et al., 2014), and serotonin (Müller et al., 2021) affects the morphology of presynaptic astrocytic processes (PAP). Additionally, physiological changes induced by fear memory formation (Badia-Soteras et al., 2023), caloric restriction (Popov et al., 2020), and long-term potentiation (LTP) (Henneberger et al., 2020) also alter PAP morphology in astrocytes. An increase or decrease in the volume of PAP impacts NMDAR-mediated activity regulation (Popov et al., 2020). Overall, this highlights the various facets by which astrocytes regulate homeostatic neuronal excitability. Dysregulation in astrocytic function could lead to a loss of E/I balance and hyperexcitability, resulting in the manifestation of brain disorders.

### 4.2 Astrocyte dynamics in physiological behavioral states

Astrocyte mediated regulation of synaptic transmission via neurotransmitter fine tuning, underlies the neuronal activity dynamics. They respond to a wide variety of stimuli under various physiological, emotional and fear states. These regulations of neuronal activity remodel the circuitry and eventually impact the behavior and cognition at system level. Similar to how establishment of social hierarchy roots from individualist behaviors, to achieve higher social orders animal displays social dominance which is modulated at a cellular level by E/I balance in dorsal prefrontal cortex (Tan et al., 2018; Wang et al., 2011; Zhang et al., 2022). Considering the role of astrocytes in maintaining E/I balance and modulating synaptic transmission, it is plausible that alterations in astrocyte function could significantly influence the processes underlying social behavior. Activity in astrocytes of globus pallidus region, of the basal ganglia participates in selection strategies in rewards seeking behaviors (Kang et al., 2023). Interestingly astrocytic network in hippocampus regulates classical hippocampal dependent functions like contextual fear memory (Badia-Soteras et al., 2023; Li et al., 2020; Orr et al., 2015), spatial memory (Hösli et al., 2022) and working memory (Nagai et al., 2021; Pinto-Duarte et al., 2019). Astrocytes have the unique ability to rapidly respond to neuromodulators and integrate signals across large populations of neurons inducing neuronal oscillations (Buskila et al., 2019). Astroglia Ca^2+^ signaling contributes to neuronal oscillations and associated cognitive functions (Lee et al., 2014). Selectively expressing tetanus neurotoxin in astrocytes reduced hippocampal gamma oscillations and impaired recognition memory (Lee et al., 2014). Apart from having a role in general behavior functions, astrocytes are particularly important in mediating abrupt state transitions during physiological functions (Van Horn et al., 2021). The ability to spatiotemporal integrate signals makes them well-suited for orchestrating these sudden shifts in brain state. Research in zebrafish has shown that astroglia can accumulate information about behavioral functions in the form of Ca^2+^ signals which further leads to behavioral state switches by activation of inhibitory neurons when astroglial Ca^2+^ levels exceed a threshold (Mu et al., 2019). Astroglia also plays a key role in sleep to wake transition which is another sudden behavioral state transition. An increase in Astrocytic Ca^2+^ signals is observed 1–2 s before the transition from slow wave sleep to wakefulness, with peak astrocytic activity observed upon awakening (Bojarskaite et al., 2020). In conclusion, these studies imply that astroglia is not just a support cell, but also a crucial player along with the neurons in modulation of physiological behavior states via regulation of optimum E/I balance and calcium homeostasis.

### 4.3 Astrocytes in epilepsy—delineating the beneficial and detrimental functions?

With the focus of AED development expanding beyond neurons, astroglial cells become particularly significant due to their integral physiological role in fine tuning neuronal activity at the tripartite synapse (Araque et al., 1999; Janigro and Walker, 2014; Oliveira and Araque, 2022). Additionally, in response to various brain insults including epilepsy, astroglial cells are known to undergo molecular, morphological and functional changes, collectively called reactive astrogliosis (Escartin et al., 2021). Astroglial cells that are crucial for homeostatic neuronal activity and neuroprotection are therefore a potential therapeutic target for neurological and neuropsychiatric disorders including epilepsy (Verkhratsky et al., 2023). However, to achieve translational goals understanding the temporal and spatial astroglial heterogeneity and how it impacts distinct disease symptoms at various stages of disease progression is essential. Deviation from a physiological to a pathological astroglial function can be beneficial or detrimental to the brain depending on the context and have been broadly classified as neurotoxic A1 and neuroprotective A2 astrocytes (Liddelow et al., 2017; Zamanian et al., 2012). The identification of similar beneficial and detrimental astrocytes, along with their unique molecular, cellular, and functional signatures at various stages of epilepsy progression such as initial brain damage from various etiologies, latency period, seizure onset, chronic epilepsy, and drug-resistant epilepsy holds significant translational relevance.

In epilepsy, astrogliosis is a well-studied disease hallmark (Patel et al., 2019; Wetherington et al., 2008), however, the heterogeneity in reactive astroglia and their beneficial versus detrimental function in various disease stages and symptoms are unclear. Astrocytes in mice models showed an increased expression of neurotrophic A2 markers in the very early time points after induction of status epilepticus; however, by 24 h there was an additional increase in the expression of neurotoxic A1 markers (Maupu et al., 2021). A study (Henrik Heiland et al., 2019) that has analyzed preexisting data derived from epileptic human hippocampal sclerosis tissue using bulk astrocyte sequencing (Zhang et al., 2016) have revealed that astrocytes in epilepsy patients have a transcriptional profile that is in between that of a typical beneficial A2 and a detrimental A1 astrocyte (Henrik Heiland et al., 2019). It is likely that these signatures arise from two functionally distinct beneficial and detrimental astrocyte subgroups, or even a hybrid astrocyte that is unique to epilepsy. A recent study using single nucleus RNA sequencing analysis has identified and characterized a subset of lipid accumulated reactive astrocytes that promote neuronal hyperactivity in both mouse epilepsy model as well as human TLE (Chen Z. P. et al., 2023). The study also identified other astrocyte subsets that had transcriptional profiles closer to beneficial astrocytes, however, this needs further functional validation. Further, unraveling the temporal and spatial dynamics of functionally beneficial and detrimental astrocyte subsets is critical in determining ideal targets for early and chronic stages of epilepsy. To achieve translational goal toward targeting astrocytes in curing human epilepsy we have to find ways to increase its beneficial functions, reduce the detrimental functions and ultimately reverse them back to physiological state. The neurotoxic A1 phenotype of astrocytes after status epilepticus was pushed toward neuroprotective A2 phenotype by silencing of circular RNA circIgf1r which was associated with a concomitant reduction in neuronal loss (Shao et al., 2021). Further studies are needed to understand how this shift from A1 to A2 astrocyte alters neuronal hyperactivity, seizures and epilepsy progression.

Timing of clinical intervention and cellular targets are also aspects that need to be considered in great detail while designing studies that are aimed at identifying drugs to reverse reactive astrogliosis toward homeostatic function. As microglial cells induce reactive astrogliosis they are considered an upstream therapeutic target to reduce reactive astrogliosis in neurological disorders with neuroinflammation (Benson et al., 2015; Henning et al., 2023; Liddelow et al., 2017). However, recent research in epilepsy has shown that targeting microglia might not have clinical relevance once reactive astrogliosis occurs (Sano et al., 2021). The researchers performed the study in pilocarpine model of epilepsy and observed that reactive phenotype occurred first in microglia and then was followed by astroglia which resulted in higher astroglial Ca^2+^ signals and increased seizure susceptibility. They further demonstrated that pharmacological inhibition of microglial activation in the very early phases prevented reactive astrogliosis and reduced seizure susceptibilities; however, in late stages, blocking microglial activation was ineffective, indicating that astroglia may need to be targeted more directly once they acquire reactive status. In the context of post-traumatic epilepsies, the timing of therapeutic interventions targeting astrocytes to prevent epilepsy is another challenging area, as reactive astrocytes in the early phases of injury also have neuroprotective functions (Linnerbauer and Rothhammer, 2020). Ablating proliferating reactive astrocytes after moderate injury increased neuronal degeneration indicating that reactive astrocytes play essential roles in preserving neural tissue and restricting inflammation after moderate focal brain injury (Myer et al., 2006). A recent study using temporal transcriptomic analysis after spinal cord injury identified that local mature astrocytes dedifferentiate, proliferate, and undergo persistent downregulation of molecules associated with astrocyte-neuron interactions, while upregulating molecules linked to wound healing, microbial defense, and interactions with stromal and immune cells (O’Shea et al., 2024). A similar process potentially facilitates the formation of neuroprotective borders in traumatic brain injuries that eventually progresses to epilepsy. Thus, in summary strategies for therapeutic modulation of reactive astrogliosis in preventing and curing epilepsy, must carefully evaluate the timing and targets of treatments to preserve their neuroprotective benefits while mitigating the detrimental contributions.

### 4.4 Astrocytes in inducing epileptiform activity

Reactive astroglia also plays a key role in shifting the balance of neuronal electrical activity leading to seizure initiation (Devinsky et al., 2013). Molecular changes that occur to expression of neurotransmitter receptors, ion channels and gap junction within the astroglia impairs their efficiency in buffering K^+^ ions and neurotransmitters in large spatial domains (Vezzani et al., 2022; Wetherington et al., 2008). Altered expression of astroglial gap junctions have been shown both in mouse and human epilepsy (Mylvaganam et al., 2014), and are implicated as a promising therapeutic target in human neocortical epilepsy (Dossi et al., 2018b). Reactive astroglia induced by virus transduction reduced inhibitory synaptic currents, due to failure in the astrocytic glutamate-glutamine cycle and downregulation of glutamine synthetase. This loss of inhibition led to hyperexcitability in hippocampal circuits, suggesting that reactive astroglia alone can cause local synaptic perturbations (Ortinski et al., 2010). However, how all of these molecular changes lead to spontaneous synchronous neuronal hyperactivity and sudden intermittent manifestation of seizures is unclear.

**FIGURE 3:**
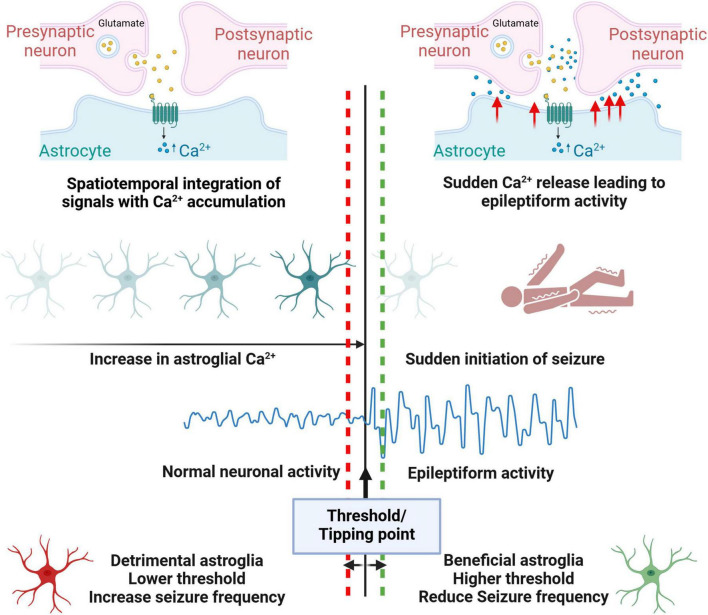
Model proposing astroglia’s role in inducing intermittent epileptiform activity. Similar to physiological contexts, in epilepsy patients, astroglia might accumulate spatiotemporal Ca^2+^ signals, reflecting a non-homeostatic state. When these signals cross a certain threshold, they can trigger seizure onset through the sudden release of Ca^2+^, causing neuronal hyperexcitability. Beneficial astroglia may raise this threshold, reducing seizure susceptibility, while detrimental astroglia lowers the threshold, making seizure initiation more likely.

With pathological cellular and molecular changes in the epileptic brain, it’s an enigma that aberrant electrical activity and seizure occurs only intermittently. Recent studies have suggested that astrocytes might play a pivotal role in sudden seizure initiation. These studies looked at the time point that leads to seizure onset and observed surges in astroglial Ca^2+^ waves prior to seizure activity both in mouse and zebrafish models (Diaz Verdugo et al., 2019; Tian et al., 2005). Surges in Astrocytic Ca^2+^waves result in glutamate release and thereby induced seizure activity (Tian et al., 2005). In zebrafish models, the transition to generalized seizures is characterized by heightened glial synchronization, massive extracellular glutamate release, and abrupt increases in neural activity and connectivity, suggesting a crucial role for glial networks in seizure generation (Diaz Verdugo et al., 2019). In our recent study in human MTLE (Ammothumkandy et al., 2022), we observed a strong anti-correlation between immature astroglia activity and circuit-level neuronal hyperactivity in the presence of 4-Aminopyridine, a K^+^ channel blocker that is commonly used to induce epileptiform activity. This striking anti-correlation closer to all or none neuronal vs astrocytic synchronized activity suggests a functional role for immature astroglia in human epileptiform activity. We hypothesize that in seizure inducing milieu immature astroglia is attempting hard to prevent excessive excitatory neurotransmission and limit neuronal hyperactivity in granule neurons; however, once calcium uptake and/or glutamate uptake exceeds a certain threshold (Eid et al., 2008; Tian et al., 2005) immature astroglia would fail at a massive scale resulting in a sudden synchronized seizure response (Figure 3). Understanding what are the key cellular and molecular events that occur in astroglia during glial synchronization and abrupt seizure initiation is therefore necessary to understand how a seizure is triggered. Better understanding of astroglia’s role in inducing abrupt state transitions during physiological functions (Buskila et al., 2019; Van Horn et al., 2021) may provide insights into how similar abrupt state transitions occur during seizure initiation. Similar to surges in astroglial Ca^2+^ waves prior to seizure (Diaz Verdugo et al., 2019; Tian et al., 2005), transient increase in Ca^2+^ waves are observed prior to physiological neuronal oscillations which benefits cognitive functions in awake mice (Lee et al., 2014). Accumulation of Astrocytic Ca^2+^ signals are linked with transition from sleep to wakefulness, (Bojarskaite et al., 2020), and sudden behavioral changes during wakefulness (Mu et al., 2019). Similar mechanisms might contribute to seizure initiation in epilepsy, where astroglia accumulate molecular signals reflecting a non-homeostatic state, which, upon crossing a certain threshold, triggers seizure onset. Whether the reactive astroglial state acts as a compensatory disease-associated protective mechanism that raises this threshold or represents a dysfunctional state with a lower threshold for seizure initiation, needs to be understood (Figure 3).

### 4.5 Astroglia a potential disruptor of sleep oscillations that lead to epilepsy

**FIGURE 4:**
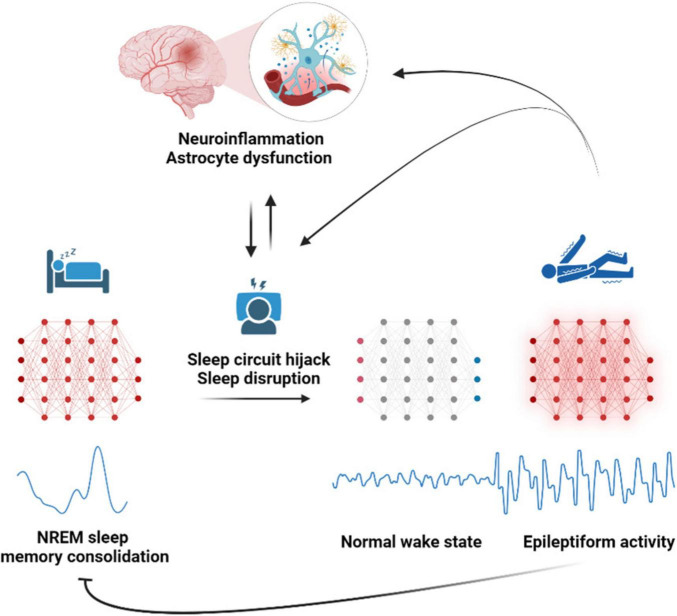
Astroglia a potential disruptor of sleep oscillations that lead to epilepsy. Astrocyte dysfunction disrupts sleep-linked brain rhythms, resulting in an increased frequency of slow-wave sleep state transitions and sleep spindles during NREM sleep. This prolonged and disrupted non - rapid eye movement (NREM) sleep compromises memory consolidation and potentially facilitates the hijacking of circuits of synchronized neuronal oscillations into epileptiform circuits, leading to seizures and epilepsy. Epilepsy further perpetuates the vicious cycle of neuroinflammation, astrocyte dysfunction, and sleep disruption, worsening the epilepsy progression.

Highly synchronized spontaneous neuronal activity oscillations during physiological NREM sleep states, particularly the slow wave sleep cycle and sleep spindles are proposed to be hijacked during pathology to act as a substrate for generation of aberrant epileptiform activity (Beenhakker and Huguenard, 2009; Mendes et al., 2021). Epileptiform activity is detected more frequently during sleep and is used in the clinic for reliable identification of epileptogenic area (Frauscher and Gotman, 2019; Sammaritano et al., 1991). As epilepsy disrupts the normal sleep cycles and the associated neuronal oscillations, cognitive functions like memory consolidation become compromised (Frauscher and Gotman, 2019; Lambert et al., 2020). The disrupted sleep cycles lead to poor sleep hygiene which further exacerbates epileptiform activity, and seizures (Badawy et al., 2006; Malow, 2004), and this continues in a vicious cycle (Krutoshinskaya et al., 2024). The role of astroglia in disrupting and hijacking sleep waves in epilepsy is unknown. During physiological sleep, astrocytic coverage of synapse reduces (Bellesi et al., 2015), potentially facilitating glutamate spillover and synchronized slow wave sleep cycle. Impairing astrocytic Ca^2+^ signaling pathway disrupts sleep linked brain rhythms resulting in an increased frequency of slow wave sleep state transitions and sleep spindles (Bojarskaite et al., 2020). Therefore, it’s likely that severely dysfunctional astrocytes in epilepsy have a similar role in sleep hijack which fuels epilepsy progression. Sleep disruption in epilepsy patients is also proposed to feed into the positive feedback loop of neuroinflammation and epilepsy progression (Bonilla-Jaime et al., 2021). Astrocytic phagocytosis, particularly of presynaptic components, increases with acute and chronic sleep loss, while chronic sleep restriction also primes microglia for activation (Bellesi et al., 2017). In response to external inflammatory signals astrocyte adenosine signaling pathway increases sleep pressure which is denoted by more of NREM sleep time involving highly synchronized neuronal slow wave activity (Nadjar et al., 2013). The neuroinflammatory milieu during various brain insults might also potentially induce sleep pressure through similar astrocytic signaling pathways, resulting in increased time spent in NREM sleep. This prolonged and disrupted NREM sleep might further facilitate the hijacking of synchronized neuronal oscillations, potentially contributing to epileptogenesis (Figure 4).

### 4.6 Astroglia in epilepsy associated cognitive decline

Biological research of cognitive psychology has throughout history mainly focused on the role that neurons play in cognitive output (van den Heuvel and Sporns, 2013). Research on the direct functional role of astroglia in cognition is still in its nascent stage (Adamsky et al., 2018; Doron et al., 2022; Kol et al., 2020; Santello et al., 2019; Sardinha et al., 2017). Astrocytic activation enhanced learning and memory by increasing neuronal activity in a task-specific way, whereas directly increasing neuronal activity resulted in external stimulus independent non-selective neuronal activity that impaired memory (Adamsky et al., 2018). Therefore, astroglia are key players in refining neuronal activity in stimulus dependent way and thereby an active player in cognition. Apart from modulating local neuronal activity, inhibiting astrocyte gliotransmitter release can impair long distance hippocampal-prefrontal theta wave synchronization and its associated cognitive functions (Sardinha et al., 2017). Reducing astrocytic vesicular release led to reduced gamma oscillations in the brain and deficits in recognition memory, highlighting the role of astrocytes in information processing and cognitive behavior by modulating brain oscillations (Lee et al., 2014). Furthermore, astrocytes themselves encode spatial information of rewards locations which is reflected by their calcium activity in familiar environments compared to new locations (Doron et al., 2022). These accumulating evidence from recent research demonstrate that astrocytes have a direct role in modulating cognition by fine-tuning task-dependent neuronal activity, facilitating long-distance synchronization of neuronal activity, and even encoding memories akin to neurons.

Similar to how research on neurons has dominated the study of physiological cognitive functions, cognitive decline in pathological conditions is mostly attributed to non-homeostatic cellular changes that drive neurodegeneration. However, these aberrant cellular changes such as autophagy dysfunction, mitochondrial dysfunction, cellular senescence, epigenetic changes, inflammation, and lipid dysregulation, not only affect neurons directly, but are also mediated indirectly by the similar changes that occur in supportive glial cells (Gonzales et al., 2022). Apart from augmenting cognitive decline by neurodegeneration, dysfunctional astrocytes have also shown to affect cognition by directly modulating neuronal networks (Habbas et al., 2015; Lee et al., 2014; Licht-Murava et al., 2023; Orr et al., 2015; Santello et al., 2019). The activation of neuroinflammatory cytokine tumor necrosis factor-α receptor in astrocytes impaired contextual memory formation in multiple sclerosis mouse models by triggering aberrant excitability of hippocampal neurons (Habbas et al., 2015). Dysregulation of astrocytic Transactivating response region DNA binding protein 43, a pathology prevalent in dementia causes progressive memory loss by modulating antiviral pathways and promoting neuronal hyperexcitability (Licht-Murava et al., 2023). Boosting astrocyte Ca^2+^ signaling in the dysfunctional astrocytes in mouse models of depression improved cognitive abilities, while it had negative effects on cognition in control mice (González-Arias et al., 2023). Thus, similar to neurons, fine-tuned levels of astrocyte activity are essential for homeostatic brain function and optimal cognitive performance. Dysfunctional astrocytes, therefore, are emerging as crucial contributors to cognitive dysfunction in pathology by directly altering neuronal activity and neural networks.

Human astroglia compared to mouse astroglia has enhanced abilities toward promoting cognition. Human embryonic glial progenitor cells, when transplanted in mice formed functional mature astrocytes that improved the learning abilities of mice (Han et al., 2013). Therefore, dysfunctional astrocytes in the human compared to mice might have a greater role toward increasing the vulnerability to cognitive decline. Human astrocytes undergo molecular changes with neurological disorders (Dossi et al., 2018a; Liddelow et al., 2017), however, their contribution to initiation and progression of cognitive decline is unclear and is a slowly evolving field of research with correlational evidence emerging with respect to astroglial reactivity. Quantification of astroglial reactivity marker Glial fibrillary acidic protein (GFAP) using Enzyme-linked immunosorbent assay in postmortem brain tissue of dementia patients showed a negative correlation with cognitive function (Kashon et al., 2004). In healthy older individuals and adults with symptomatic Alzheimer’s disease higher serum levels of GFAP associated with worse memory performance (Bettcher et al., 2021). A study in cognitively unimpaired Amyloid beta positive individuals has shown that serum levels of GFAP, can predict development of Tau pathology, which has the likelihood of progressing into Alzheimer’s disease with cognitive impairment (Bellaver et al., 2023). In light of recent evidence for a direct astroglial role in physiological cognitive functions (Santello et al., 2019), and the molecular changes astrocytes are known to undergo during epileptogenesis (Vezzani et al., 2022), a direct link between astrocyte dysfunction and cognitive decline in epilepsy is highly suggested. However, further studies are needed to establish this connection and to understand epilepsy-specific cognitive modulation by astrocytes, which is critical for developing therapeutic strategies to slow, prevent, or potentially reverse cognitive decline in epilepsy patients.

### 4.7 Astroglia in epilepsy associated mental health comorbidities

Evidence for the direct cellular role of astroglia in modulating mental health is limited and has started to emerge from rodent studies with selective astroglial targeting. Experimental depletion of GFAP+ astroglia in the prefrontal cortex of rodents induces anhedonia-like behaviors, while enhancing their activity reverses these behaviors, highlighting the critical role of cortical astroglia in depression (Codeluppi et al., 2023). In a transgenic mouse model with [disrupted-in-schizophrenia 1 (DISC1)-N] mutation, that induces impaired risk assessment response, researchers activated astrocytes in the basolateral amygdala and consequently restored normal behavior (Zhou et al., 2024). The aberrant astroglial changes in human mental health disorders and their therapeutic targeting is an even more challenging area of investigation due to the complex categorization of these disorders and their frequent co-occurrence with other neurological conditions. Mental health conditions can generally be categorized into affective disorders (e.g., major depressive disorder MDD), anxiety disorders (generalized anxiety disorder GAD) psychotic disorders (i.e., schizophrenia, bipolar disorder type I, schizoaffective) and stress-related disorders (post-traumatic stress disorder, PTSD) (Atwoli et al., 2015; Karagianis et al., 2009; Steel et al., 2014; Vollebergh et al., 2001). Studies in post-mortem human tissue of patients with MDD have demonstrated lower expression of astrocyte markers GFAP and vimentin as well as decreased astrocyte density notably amongst the prefrontal cortex and hippocampus (Cobb et al., 2016; Liu J. et al., 2022; O’Leary and Mechawar, 2021; Qi et al., 2019). In human post-mortem schizophrenia brains, there is heterogeneity in astrocyte morphology and levels depending on the regions of the brain (Zhang et al., 2021). Taking into consideration the critical role of astrocytes in synthesizing and secreting key neurotrophic factors, which regulate the neurogenic microenvironment, it is hypothesized that stress or trauma-induced impairment of astrocytic function may contribute to the pathophysiology of PTSD (Li et al., 2022). In a rat model of PTSD, the hippocampus showed significant decline in GFAP positive astrocyte densities (Saur et al., 2016). Although, in another study utilizing a foot shock rat model of PTSD, there was an increase in reactive astrogliosis evidenced by histologically higher levels of S100β, and then a decline in s100β upon administration of anti-depressant ketamine (Valenza et al., 2024). These studies collectively demonstrate a huge variability in astrocyte alterations in mental health conditions and a need to identify the specific changes associated with each neurological disorder.

Epilepsy patients disproportionately experience mental illness comorbidities (Berg et al., 2017; Tsigebrhan et al., 2023; Uepping et al., 2021). Mood and anxiety disorders are amongst the most common mental illness comorbidities affecting roughly between 25% and 35% of epilepsy patients (Lu et al., 2021). The reported prevalence for PTSD comorbidities in epilepsy patients is 14.2% and for psychosis/schizophrenia it is about 7.4%. There are limited studies that have addressed astrocyte changes in association with mental health co-morbidities in epilepsy patients. In patients with MTLE, there was an increase in GFAP within the hippocampus among those with a history of interictal psychosis compared to those with no psychiatric history or a history of major depression (Kandratavicius et al., 2015). Additionally, an investigation into astrogliosis in three MTLE patient groups – (1) without mental disorders (2) with mental disorders (3) with depression specifically, revealed that those with mental disorders or depression exhibited greater neuronal loss and astrogliosis. Interestingly, the expression of astrogliosis markers presented a more complex pattern with GFAP and aquaporin-4 (AQP4) levels being lower in MTLE patients with mental disorders and depression specifically, whereas Metallothioneins I/II (MT-I/II) levels being higher (Lu et al., 2019). It is unclear whether these changes are mediated by mental health co-morbidity or the associated therapeutic treatment. Drugs that are used to treat mental health conditions have shown to alter astrocyte functions, potentially mediating the therapeutic benefits (Koyama, 2015). An in-depth investigation of these clinical variabilities and their role in epilepsy progression is warranted to identify more targeted molecular modulations of astrocytes among patients with and without mental illness comorbidities.

## 5 Discussion/concluding remarks

In this review we connect the literature on spontaneous synchronized neuronal circuits, their physiological roles and their aberrant forms in epilepsy to understand how knowledge of cellular players in physiological functions can enhance our understanding of how they get hijacked during disease leading to seizures and cognitive decline. The nervous system functions by stimulus evoked, fine-tuned, spatially restricted neuronal activity. However, spontaneous non-stimulus dependent neuronal hyperactivity occurs in few physiological states such as neurodevelopment, memory consolidation during NREM sleep cycle and free memory recall during wakefulness. In pathological conditions, aberrant spontaneous synchronized neuronal hyperactivity called epileptiform activity results in debilitating seizures. In addition to seizures, epilepsy patients also undergo progressive cognitive decline. Therefore, there is an urgent need to identify mechanisms and therapies that modulate aberrant neuronal hyperactivity in the human brain. Despite the development of approximately 30 approved anti-seizure medications, achieving seizure freedom has shown limited improvement. This warrants identification of new cellular targets for epilepsy treatment. With the focus of anti-epileptic drug development expanding beyond neurons, astroglial cells become particularly significant due to their integral physiological role in fine tuning neuronal activity at the tripartite synapse, recently emerged role in large scale physiological neuronal circuits, modulation of behavior, as well as their role in neuroinflammatory pathways and epileptogenesis. We discuss astroglia’s (1) transition from a physiological to pathological state during epilepsy, (2) beneficial vs detrimental role in epilepsy (3) role in disrupting physiological circuits to form epilepsy circuits (4) role in integration of non-homeostatic signals leading to intermittent seizure initiation (5) role in cognitive and mental health co-morbidities by modifying neural circuits and facilitating neurodegeneration. Understanding how astroglia integrate signals to create spontaneous neuronal activity in physiological functions and how it evolves with disease could provide novel therapeutic targets for epilepsy as well as other pathological conditions with aberrant neuronal circuits.
